# Ethical Lessons from an Intensivist’s Perspective

**DOI:** 10.3390/jcm11061613

**Published:** 2022-03-15

**Authors:** Jean-Louis Vincent

**Affiliations:** Department of Intensive Care, Erasme Hospital, Université Libre de Bruxelles, 1070 Brussels, Belgium; jlvincent@intensive.org

**Keywords:** distributive justice, proportionality, withholding, rationing, intensive care, COVID-19, communication

## Abstract

Intensive care units (ICUs) around the world have been hugely impacted by the SARS-CoV-2 pandemic and the vast numbers of patients admitted with COVID-19, requiring respiratory support and prolonged stays. This pressure, with resulting shortages of ICU beds, equipment, and staff has raised ethical dilemmas as physicians have had to determine how best to allocate the sparse resources. Here, we reflect on some of the major ethical aspects of the COVID-19 pandemic, including resource allocation and rationing, end-of-life decision-making, and communication and staff support. Importantly, these issues are regularly faced in non-pandemic ICU patient management and useful lessons can be learned from the discussions that have occurred as a result of the COVID-19 situation.

## 1. Introduction

The pandemic caused by severe acute respiratory syndrome coronavirus 2 (SARS-CoV-2) has had an enormous impact on individuals, families, organizations, economies, and so much more. As of 8 March 2022, more than 440 million cases of SARS-CoV-2 infection have been officially reported and more than six million deaths (https://coronavirus.jhu.edu, accessed on 11 February 2022). Intensive care units (ICUs) worldwide have been particularly involved in caring for patients with severe infection (coronavirus disease 2019 (COVID-19)), a situation that, in addition to the clinical challenges, has created or intensified the debate around important ethical questions.

The sudden, relatively unexpected influx of large numbers of severely ill patients, many of whom required ventilatory support, stretched intensive care facilities and staff to their limits and meant that triaging patients or rationing resources was necessary for many units. Moreover, in line with the general population, many staff members became infected, had to isolate because of high-risk contacts, or needed to care for vulnerable family members, leading to staff shortages and thus resulting in further limitations of resources and the need to transfer staff with little training or preparation from other units. Difficulties in communicating with patients because of personal protective equipment made patient care more complex and less personal and the fact that no visitors were allowed further increased the burden of care on staff members.

In this review, we will discuss some of the major ethical aspects of the COVID-19 pandemic from an intensive care perspective.

## 2. Resource Allocation and Triage

Intensive care is expensive and hospital managers, ICU directors, and individual doctors are all faced with difficult choices on a regular basis as demand for intensive care services increasingly surpasses supply. But in the surge of the pandemic, the already fragile (in many countries and units) demand/supply balance in healthcare systems as a whole, but particularly in the ICU, was exacerbated by the huge, sudden, and prolonged influx of patients. Indeed, this surge in patient numbers was associated with increased mortality as reported recently in a retrospective study of 144,116 patients with COVID-19 admitted to 558 hospitals in the USA, in which approximately 25% of deaths were attributed to the sudden rise in patient numbers [1].

Admissions to the ICU were, therefore, sometimes difficult to obtain, not only because of (often long-standing) ICU bed shortages but also the lack of equipment and especially personnel [2]. Attempts were made to reorganize hospitals to increase the ICU bed availability by converting other areas, such as post-operative care units, into temporary ICUs, facilitated by reducing the admissions for non-COVID-19 patients, which also helped free staff for relocation to COVID-19 units [3,4]. In some countries, large field hospitals were constructed as new buildings or within repurposed gymnasia or conference halls [5]. However, extra beds require more equipment and additional staff, with the risk that the quality of patient care will be reduced if staffing is not sufficient in numbers or the degree of training [6]. In this situation, rapid but adequate training must be provided (a number of online courses are now available [7]) and inexperienced nurses and doctors should be supervised by those with experience. In Table 1, some of the methods that have been used to cope with ICU shortages are summarized.

When it was not possible to increase the ICU capacity or transfer patients to hospitals or ICUs that still had capacity, difficult choices sometimes had to be made regarding which patients could or should be admitted, to try and ensure admission and treatment for those most likely to survive with a good quality of life, as should also be the case during non-COVID-19 times [8]. With the very high numbers of patients suddenly requiring intensive care, and the lack of information about patient risk, the disease course, and likely outcomes, it was, however, difficult to establish triage protocols, and some centers initially adopted a ‘first come, first served’ strategy to decide who should and should not be admitted. However, ICU admission criteria should “direct limited resources toward patients most likely to benefit from them” [9], with the equal and fair allocation of available resources to all. A first-come, first-served policy does not meet this ethical principle of distributive justice as patients who become ill later in the course of the pandemic would receive worse care than those who are ill early in the pandemic or who have easier access to healthcare [10]. Similarly, allocating by a “lottery” cannot be justified as no two patients are identical and the problem is not just the lack of an available ICU bed but also the lack of necessary equipment and staff.

Multiple protocols and guidelines for triage, from local, national, and international groups, have been published [9,11,12,13,14]. As in non-pandemic conditions, age, degree of frailty, comorbid conditions, and previous organ dysfunction, are the four most important criteria to help predict patient survival when making triage decisions for COVID-19 patients [9,11,12,13,14]. These have been combined with other factors in various “prediction of survival” scoring systems for patients with COVID-19 [15]; however, frequent recalibration is likely to be necessary as the pandemic evolves, and although such scores can help inform decisions, clinical judgment should remain an essential component of triage [16,17]. Importantly, the person’s preferences must also be taken into account whenever possible, although they may not be known in the intensive care environment if advanced care planning has not previously been discussed or documented.

It has been proposed that, in the context of a pandemic, all admissions to the ICU and interventions that are started should be considered as “time-limited trials” based on pre-defined treatment goals [18], as has been suggested outside of COVID-19 when patient outcomes are not certain [19,20]. If the treatment goals are not met within a set time, and it is considered that further or continued intervention is futile or disproportionate, supportive therapies could then be withdrawn and/or the patient discharged from the ICU so that resources can be re-allocated to give others a chance to benefit [21]. This decision must be clearly documented and, whenever possible, discussed with the patients and their next of kin before proceeding [22].

COVID-19 triage committees may be helpful in some situations [23,24], perhaps particularly in developing triage guidelines at the local level [25], but they may create an unnecessary delay at the bedside in the critical pandemic situation [25,26].

### The Special Case of Extracorporeal Membrane Oxygenation

Extracorporeal membrane oxygenation (ECMO) has been used in more than 11,000 severely ill adult patients with COVID-19 since the start of the pandemic. The ICU mortality of these patients is 47%, as reported in the Extracorporeal Life Support Organization (ELSO) Registry (www.ELSO.org, accessed on 11 February 2022). However, while ECMO may benefit some patients with severe acute hypoxemic respiratory failure, it is a highly resource-intensive mode of support in terms of equipment, staff, and the time for set-up and monitoring. Development of local and national ECMO networks may be helpful to coordinate and optimize the allocation of ECMO resources in pandemic conditions [27], but when healthcare systems are overloaded, use of this resource-intense therapy may be seen as against the tenet of distributive justice, with decisions made accordingly to restrict its use so as not to reduce the quality of care for other patients [28].

## 3. End-of-Life Decision Making

End-of-life decisions form a regular part of ICU practice, but have become more frequent and perhaps even more challenging during the COVID-19 pandemic with the large numbers of patients and high death rates, in addition to complications associated with reduced family visits and thus difficulty communicating with family members. Indeed, during surges in patient numbers, most ICU patients die because of withholding (of ICU admission, mechanical ventilation, ECMO) [29]. As with the triage considerations mentioned earlier, the proportionality of care must take precedence when deciding whether to withdraw or withhold life-sustaining treatment, at any time, pandemic or not. Interventions should only be continued if their likely benefit outweighs any potential harm. Life-sustaining interventions should not be continued if the patient has no chance of quality survival [30]. The same as during non-pandemic periods, patients at the end of life should be kept comfortable and allowed to die with dignity; this may entail using analgesics/sedatives, even if such agents may shorten the dying process [30]. Patients’ preferences should again be taken into account when making such decisions and, where possible, these difficult decisions should be made in concert with family members.

## 4. Communication and Support

The relationship between the healthcare team and patients’ relatives during the pandemic was sometimes (often) suboptimal, especially in the earlier waves when hospitals were stretched to their limits and visiting was highly restricted or forbidden. Relatives received news intermittently by telephone and often did not see their loved one between admission and discharge or death. Rapidly, the use of video links was adopted wherever feasible so that patients and relatives could talk if possible or at least have visual contact. However, although virtual visiting can reduce patient and relative stress and improve staff morale, it cannot replace human contact and is not without challenges including lack of staff time, difficulties some individuals have with operating technology, and lack of access to devices or an internet connection [31,32].

This inability for relatives to accompany the seriously ill or dying person places considerable additional stress on the family members. Relatives of critically ill patients are known to be at risk of depression, anxiety, and post-traumatic stress disorder [33,34,35], and the enforced lack of personal contact during the current pandemic is likely to have increased this risk. Good communication with relatives is essential to try and mitigate some of this risk [36]. In this context, Azoulay and Kentish-Barnes [37] have suggested a five-point strategy to try and optimize communication during COVID-19: allowing (restricted) family visits where possible; providing written information that can be shared among family members; scheduling regular telephone/video link updates between a staff member and a selected family member; encouraging the family to stay in touch with the patient using diaries, drawings, video calls etc. where possible; increasing communication and enable more extensive visiting if the patient is at the end of life. Importantly, establishing and maintaining good communication with relatives will also help to reduce the burden on the healthcare team.

## 5. Psychological Impact on Healthcare Workers

The unusually heavy and stressful workload during the pandemic has had a major impact on the mental health of many healthcare workers. ICU medical and nursing staff were faced with an unprecedented number of patients, many of whom did not survive. The sheer numbers of patients, as well as the time needed to put on and remove personal protective equipment, meant that the time available for each patient was inevitably less than normal, and many staff members felt they were unable to provide the quality of care that they would under non-pandemic conditions. Lack of effective treatments, difficulties communicating with patients and their relatives, the need to make rapid, complex ethical decisions regarding which patients to admit or when to withhold or withdraw treatment, responsibility for new, non-ICU-trained members of the team transferred from other units, and fears about catching the virus and passing it on to their own family members, all added to the psychological burden on ICU teams with high rates of insomnia, depression, anxiety, post-traumatic stress disorder, and burnout [38,39,40,41,42,43]. Various strategies have been proposed to try and limit some of the psychological impact, including adequate training and senior support for new or re-allocated staff, availability of clinical psychologists for individual assessments and consultation, and regular team debriefing and support sessions [44,45]. Psychological support should be offered openly, with a low threshold for intervention, and be easily accessible. Requesting psychological support is still seen by many as a sign of weakness [46] and every attempt must be made to ensure those asking for or receiving psychological support are not stigmatized for doing so [47,48]. Moreover, many of these symptoms are likely to last long after the COVID-19 pandemic has eased or disappeared so long-term and continuing follow-up support is going to be important. Importantly, non-medical staff may have even higher rates of psychological distress [49] and must be included in any psychological support offered. 

## 6. Impact on Non-COVID-19 Patients

**Figure 1:**
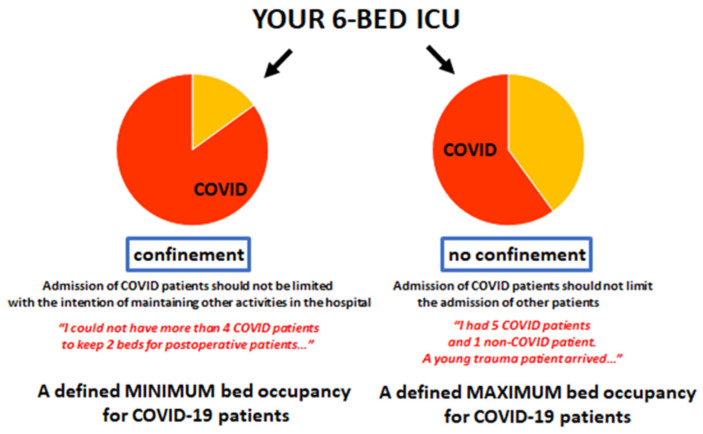
Two phases of reserving ICU beds for COVID-19 patients.

The fact that a pandemic is ongoing has no effect on the development of other disease processes, and urgent treatments should not be impacted by the surge in patient numbers or the limitations imposed by hospital reorganization to increase capacity for COVID-19 patients. When triage is necessary, resources must be shared equally for patients with and without COVID-19. Nevertheless, during the COVID-19 pandemic, diagnosis and treatment of non-COVID-19 acute and chronic conditions has decreased [50,51,52,53], partly because many hospital units were closed, meaning routine hospital practice, such as elective surgical interventions, was suspended to reduce the pressure on ICU beds, or because these beds were repurposed, and also because patients delayed making or attending appointments for fear of catching COVID-19 or of further burdening the healthcare system. Indeed, there were two periods in many ICUs during the surge in admissions with a proportion of ICU beds reserved specifically for patients with severe COVID-19 during those peaks, partly to ensure that all hospitals treated these patients equally. Later, as hospitals re-opened, this proportion was reduced to enable more non-COVID-19 patients to be managed in the ICU (Figure 1). Nevertheless, it is anticipated that there will be a high burden of excess morbidity and mortality deaths over the next years due to delayed or inadequate treatment of acute and chronic conditions.

## 7. Conclusions

The SARS-CoV-2 pandemic has made us realize how poorly equipped healthcare systems worldwide are to deal with such a massive influx of severely ill patients. Many ethical issues, most of which were already present pre-pandemic, have come to the forefront, forcing rapid reflection and leading to the establishment of new guidelines and protocols, which should be built on for use in regular ICU practice but also in preparedness for pandemics of the future. Ensuring resources are allocated equally without discrimination, that patient management is proportional, that quality communication is maintained between the staff and patient, staff and relatives, and patient and their family, and that psychological support is available for all staff are key to the effective, efficient functioning of the ICU during and post-pandemic.

## Figures and Tables

**Table 1:** Some methods used to try and cope with the shortage of ICU facilities.

**Space**	Use all ICU beds that can be made available—expand into the cardiac care unit, post-anesthesia care unit/recovery room, etc. Create intermediate care units in nearby areas
**Staff**	Welcome nurses and doctors from other departments Use a pyramidal approach to ensure adequate supervision of those with less training/experience Organize rapid training programs
**Stuff**	Consider using anesthesia respirators Consider using one respirator for two patients
**Systems**	Establish clear rules/scores for admission/discharge Admit the need to withhold/withdraw therapy for some patients

## Data Availability

Not applicable.
